# Dynamic Interplay Between Wake Slow Waves and Epileptiform Discharges in the Epileptogenic Zone

**DOI:** 10.1212/WNL.0000000000214001

**Published:** 2025-08-20

**Authors:** Laurent Sheybani, Umesh Vivekananda, Nishant Sinha, Erin Conrad, Brian Litt, Neil Burgess, Daniel Bush, Matthew C. Walker

**Affiliations:** 1 Department of Clinical and Experimental Epilepsy, UCL Queen Square Institute of Neurology, University College London, United Kingdom;; 2 National Hospital for Neurology and Neurosurgery, University College London Hospitals NHS Foundation Trust, United Kingdom;; 3 NIHR University College London Hospitals Biomedical Research Centre, United Kingdom;; 4 Now with Department of Clinical Neuroscience, Faculty of Medicine, University of Geneva, Switzerland;; 5 Department of Bioengineering, School of Engineering and Applied Sciences, University of Pennsylvania, Philadelphia;; 6 Center for Neuroengineering and Therapeutics, University of Pennsylvania, Philadelphia;; 7 Department of Neurology, Perelman School of Medicine, University of Pennsylvania, Philadelphia;; 8 Institute of Cognitive Neuroscience, University College London, United Kingdom; and; 9 Department of Neuroscience, Physiology and Pharmacology, University College London, United Kingdom.

## Abstract

**Background and Objectives:**

Outcome of epilepsy surgery remains suboptimal, calling for the identification of new, complementary biomarkers of the epileptogenic zone (EZ). Recently, we identified local wake slow waves (LoWS) as a potential regulator of network excitability that interacts with interictal epileptiform discharges (IEDs). In this study, we tested whether this interaction is associated with surgical outcome.

**Methods:**

In this retrospective study, we analyzed intracranial recordings from patients with intractable focal epilepsy who underwent surgery at the Hospital of the University of Pennsylvania. We used surgical success as an indicator that most or all of the EZ had been resected. We used linear mixed models to test whether the incidence of IEDs and LoWS, as well as their interaction, can accurately delineate the EZ in patients with successful vs poor outcome.

**Results:**

Across 55 patients (30 women, mean age ±SD: 34 ± 10 years), we found that, although IEDs were more frequent in the seizure-onset zone, their rate in resected and nonresected areas was not associated with surgical success. Indeed, neither the rate of IEDs (F [1, 52.57] = 0.070, *p* = 0.793) nor that of LoWS (F [1, 48.81] = 1.1032, *p* = 0.299) in resected vs nonresected areas differed across surgical outcomes. Next, we examined their interaction, validating our previous findings in this larger, independent cohort, by confirming that the closer the LoWS are to an IED, the lower the network excitability during the IED. Furthermore, we found that the delay from IED to the subsequent LoWS—but not the reverse—is associated with surgical outcomes (IED to LoWS: F [1, 52.17] = 5.344, *p* = 0.025; LoWS to IED: F [1, 52.56] = 1.038, *p* = 0.313), with shorter delays observed within the EZ. We confirm this using classification analyses that yielded a significant accuracy of 63% (interquartile range: 57%–69%, *p* < 0.0001), underscoring its potential utility as an additional biomarker of the EZ.

**Discussion:**

The temporal proximity of LoWS to a preceding IED in the resected cortex is associated with surgical outcome. This may reflect changes in the regulation of network excitability in the EZ as a form of homeostatic regulation. It raises the possibility to use this index as an additional prognostic biomarker in epilepsy surgery.

## Introduction

Pharmacologic treatment fails to control seizures in up to 30% of people with epilepsy.^1^ These patients are candidates for epilepsy surgery, provided that a brain region responsible for generating seizures can be identified and that its surgical removal is not expected to cause unacceptable deficits. Presurgical evaluation of epilepsy relies on the availability of markers that can reliably identify the epileptogenic zone (EZ), that is, the region of the brain that needs to be resected to obtain seizure freedom (which may differ from the seizure-onset zone [SOZ], i.e., where seizures originate based on clinical investigations). Only 40%–50% of operated patients remain seizure free at 10 years,^2,3^ which calls for the development of new, complementary biomarkers.

Neurophysiology and structural, functional, and metabolic imaging are classic tools used to delineate the EZ in presurgical evaluation of epilepsy.^4-13^ When no obvious structural lesion is present, electrodes are often placed into or onto the brain to more accurately define the SOZ. Different markers can be combined to develop scores that help determine the focality of epilepsy.^14,15^ Typical neurophysiologic biomarkers are interictal epileptiform discharges (IEDs) and seizures, but IEDs remain a suboptimal marker of the EZ^6,7,16-18^ and confounding factors can affect their reliability to delineate the EZ. Seizures can propagate, and the real topographical onset can be missed if the intracranial electrode coverage does not sample the region generating seizures. In such cases, the first electrode exhibiting ictal activity would point toward a propagation area, rather than the actual SOZ. The fact that rapid seizure propagation (<10 seconds) is associated with a lower probability of seizure freedom after resective surgery highlights this ambiguity.^19^

Recently, we identified local wake slow waves (LoWS) in intracranial recordings of people with epilepsy.^20^ In previous work, we demonstrated that their frequency is related to that of IEDs and that network excitability during IEDs, measured by high-γ (HG) power as a proxy of neuronal activity,^21^ increases with the latency from the last LoWS in the same electrode. This suggests that LoWS reflect a compensatory process that dissipates with time.^20^ We thus hypothesized that LoWS are generated in response to IEDs as a homeostatic mechanism to counter circuit hyperexcitability. In this study, we reasoned that this interaction between LoWS and IEDs would provide a better indication of aberrant network excitability than either marker alone and could, therefore, help to delineate the EZ. Using a data set of intracranial recordings from awake patients undergoing presurgical evaluation for pharmacoresistant epilepsy,^22-24^ we first tested whether simple metrics, that is, the incidence rate of IEDs and LoWS, were associated with surgical outcome. We then examined changes in the rate of IEDs and LoWS before seizures as a further indicator of their relationship with ictogenesis. Finally, we tested whether the temporal relationship between those events was associated with surgical outcome. We demonstrate that the temporal delay between IEDs and subsequent LoWS has prognostic significance, which is not present when considering rates of IED or LoWS independently. More specifically, we found that regions that should be resected present with a shorter latency between IEDs and LoWS. Crucially, we also show that this metric can be used to accurately classify patients into those with good and poor surgical outcomes, highlighting its utility as an additional biomarker during surgical planning.

## Methods

### Standard Protocol Approvals, Registrations, and Patient Consents

Informed consent was obtained from all participants included in this study, which was approved by the Institutional Review Board of the University of Pennsylvania under Institutional Review Board (IRB) 811097.

### Hospital of the University of Pennsylvania (HUP) Data Set

The Hospital of the University of Pennsylvania (HUP) data set^22-24^ consists of 57 adults who underwent stereotactically inserted depth electrodes, subdural grid, and/or strips as part of the presurgical evaluation of epilepsy (Table 1). The data are provided with different sampling frequencies (256 Hz: 1 patient; 500 Hz: 12 patients; 512 Hz: 29 patients; 1,024 Hz: 15 patients). Nine patients had unsuccessful epilepsy surgery before acquisition of the current intracranial recordings and were included because their outcome was no different from that of nonoperated patients (Results). All but 1 patient had 2 sessions of interictal recording, each of a duration of 5 minutes, a length that is superior to that in similar studies that have focused on new markers of the EZ.^24-26^ The number of sessions for ictal recording varied from 1 to 5 with a mean ± SD duration of 7.4 ± 2.6 minutes per patient.

**Table 1:** Demographics of the Population

	Engel 1–2	Engel >2	*p* Value	Test
Age (mean, SD)	35 (10)	33 (12)	0.75	Unpaired *t* test
Biological sex (female–male)act	19–23	10–3	0.06	Fisher
Hemisphere (right–left)	19–23	4–9	0.52	Fisher
Delay (mo, median, IQ)	24 (24–24)	24 (12–24)	0.22	Mann-Whitney
Therapy (resections–ablation)	23–19	5–8	0.36	Fisher
Recording (SEEG–ECOG)	24–18	11–2	0.10	Fisher
Lesional (Y–N)	20–22	6–7	0.99	Fisher
Onset (y, mean, SD)	16 (12)	19 (15)	0.42	Unpaired *t* test
Previous operation (Y–N)	6–36	3–10	0.43	Fisher

Demographics of patients included in the study. Potential confounders are equally distributed between patients with successful and poor surgical outcomes. Further details are available in eTable 1. The data set overlaps with that of Bernabei et al.^24^

### Preprocessing

Electrodes located in the gray matter were selected for analyses after exclusion of artifactual signal (eMethods and eFigure 1 provide further details). Reference was located away from the suspected EZ, usually in the medullary bone in the skull, as indicated in a previous publication using this dataset.^25^ Because the incidence of slow waves varies across the sleep-wake cycle,^20^ only awake data were included, defined by the alpha/delta ratio, as described previously.^24^ A 60-Hz notch filter was applied before analysis. More details of acquisition and preprocessing are available in a previous study using this dataset^25^ and eMethods.

### Identification of IEDs

**Figure 1:**
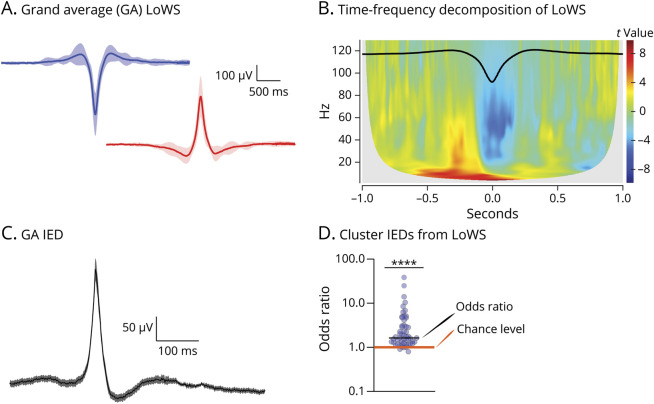
Grand Averages of Local Wake Slow Waves (LoWS) and Interictal Epileptiform Discharges (IEDs) (A) Grand average of LoWS (mean ± SD, n = 55 patients) with negative (blue) and positive (red) polarities. Among these waves, we selected only those associated with a downstate (Methods). eFigure 3 presents individual examples. (B) Time-frequency representation of selected waves. A decrease in γ to high-γ power is seen in the center of the window, confirming the presence of a downstate associated with the selected waves. The overlying black trace is the grand average of LoWS across patients. (C) Grand average of IEDs (mean ± SD, n = 55 patients). eFigure 2 presents individual examples. (D) Odds ratios of the clustering analysis between LoWS and IEDs across patients, with an odds ratio significantly above 1 (median [interquartile range]: 1.64 [1.26–3.17], one-sample Wilcoxon test, *p* < 0.0001, n = 55 patients). Black horizontal line: median. Orange horizontal line: chance level.

IEDs were identified using an automated process described in detail elsewhere.^20,27^ In brief, the data were filtered within the 20–80 Hz band, and then, the envelope was extracted using the Hilbert transform. Candidate events were selected if they had an amplitude 3.5 times greater than the mean envelope across the entire recording and discarded if their amplitude was below 4 times the mean envelope of the original signal (after 5-Hz high-pass filtering to eliminate any direct current [DC] signal offset). In this multisite intracranial data set, we added a further step that categorized IEDs into isolated events and events belonging to bursts, which were defined as sequences of IEDs occurring within 0.2 seconds. Only the first IED within a burst was taken into account to avoid including propagated IEDs. Given the large number of patients and electrodes, all automatically IEDs could not be visually checked. Representative examples (eFigure 2) and grand average of IEDs (Figure 1) were similar to typical IEDs described in intracranial EEG.

### LoWS Detection

Detection of LoWS followed the same strategy as described by Frauscher et al.^28^ and Sheybani et al.^20,29^ The signal is first filtered between 0.5 and 4 Hz; then, zero-crossings are marked; and any half-wave whose duration lasts between 0.25 and 1 second is identified as a candidate wave. Candidate waves whose amplitude is below the 90th percentile of all detected waves per electrode are discarded. Candidate waves that occur within 1 second of a detected IED on the same electrode are also discarded to avoid selecting post-IED waves, which are different electrophysiologic activities.^30^ Only waves associated with a downstate, that is, decreased HG power (HG, 45–130 Hz), were included in further analyses (eMethods provide further details).^20,31^

Hence, detection of LoWS relied on different parameters from those used in IED detection, including the use of a different frequency band (IEDs: 20–80 Hz; LoWS: 0.5–4 Hz), the latency criteria between zero-crossings that apply to LoWS, but not to IEDs, and the association of LoWS, but not IEDs, with a downstate. Several other criteria further support the fact that LoWS are different events from IEDs, which can be found in our previous publication^20^ as well as in the eMethods. Representative examples (eFigure 3) and grand average of LoWS (Figure 1) are visually different from detected IEDs. To further demonstrate that LoWS and IEDs are 2 different entities, we computed a cluster analysis based on their morphologic features (eMethods provide further detail).

### Interplay Between IEDs and LoWS

*Interaction between delay since the last LoWS and IEDs*. To test whether we could replicate the finding of an association between IED excitability and delay from the last LoWS,^20^ we computed a linear mixed model (LMM) using IED-associated excitability (estimated by HG power during IEDs, henceforth IED-HG) as the dependent variable and delay since the last LoWS as the independent covariate. We included a random intercept and random slope in the model. The model is defined by the following equation:

(1) $$IED_{HG}\;∼\;1+delay+\left(1+delay\;|\;patients\right)$$

where *IED*_*HG*_ is the IED-HG and *delay* is the delay since the last LoWS. To measure HG power during IEDs, we first extracted IEDs in individual windows of ±3 seconds centered to IEDs' peak. We then computed a Hanning taper frequency decomposition to assess HG power between −0.05 and 0.05 seconds around IEDs and log-transformed and normalized the data to baseline.

### Prognostic Value of the Different Indices

*Rates of LoWS and IEDs inside vs outside the SOZ.* To test whether the rates of LoWS or IEDs were different inside and outside the clinically determined SOZ, the SOZ was defined as the presumable region of seizure onset based on the combination of intracranial recording and scalp EEG, MRI, and nuclear medicine when available. An average of 7 electrodes (SD = 5) per patient were located in the SOZ and 62 (SD = 26) outside the SOZ; that is, 13% (SD = 12%) of electrodes belonged to the SOZ across patients.

*Prognostic value of rates of LoWS and IEDs.* To test whether the rate of LoWS and IEDs is different inside and outside the EZ, we computed 2 LMMs using the mean incidence rate of LoWS and IEDs within resected and nonresected areas as dependent variables, the resection status as a binary independent factor, and the Engel score converted into a linear scale as an ordinal covariate, following the study by Kanchanatawan et al.^32^ The equations of the respective LMM were as follows:

(2) $$rate_{IEDs}\;∼\;1+Engel+resection+Engel\times resection+\left(1\;|\;patients\right)$$

(3) $$rate_{SWs}\;∼\;1+Engel+resection+Engel\times resection+\left(1\;|\;patients\right)$$

where × denotes the interaction term between the Engel score and the resection status of the electrode.

*Prognostic value of LoWS-IED interaction.* To test whether the delay between LoWS and the subsequent IED, or the inverse, is associated with outcome, we input LoWS-to-IED and IED-to-LoWS delays, corrected for the variable incidence of these entities across electrodes (eMethods) within resected and nonresected areas as an independent variable in an LMM, together with the resection status as a binary independent factor and the Engel score converted into a linear scale as an ordinal covariate, as follows:

(4) $$corrected_{delay}=1+resection+Engel+Engel\times resection+\left(1\;|\;patients\right)$$

To assess whether any effect was dependent on cortical structure, lesional status, or type of resection, we computed another LMM with the SOZ, lesional status, and type of resection as random coefficients, as follows:

(5) $$corrected_{delay}=1+Engel+resection+Engel\times resection+\left(1\;|\;patients\right)+\left(SOZ\;|\;patients\right)+\left(lesion\;|\;patients\right)+\left(therapy\;|\;patients\right)$$

### Classification

We used the ratio of the corrected IED-to-SW delay between resected and nonresected areas per patient as a metric to classify patients with good (Engel I-II) and poor (Engel >II) outcomes. The classifier was trained on a subset of patients (“training set”) and then validated on an independent subset of the remaining patients (“validation set,” eMethods and eFigure 4). Odds ratio, accuracy (total of correctly identified patients divided by the total number of patients), F1 score (a measure of predictive performance, equation mentioned further), specificity (Sp), sensitivity (Sn), and negative and positive predictive values (NPV and PPV, respectively) are provided to assess the performance of the classifier. The F1 score is defined by

(5) $$F.1score=2.\;\frac{PPV.Sn}{PPV+Sn\;}$$

True positives were defined as patients with successful outcome who were classified as such. True negatives were patients with poor outcome who were classified as such.

### Seizure Analyses

We included as “preictal” activity the period preceding seizure detection in each individual file containing a seizure (mean ± SD duration of the preictal period across patients: 110 seconds ± 2 seconds), up to 10 seconds before seizure onset (i.e., the period from −10 seconds to the onset of seizures is not included in analyses). We then visually identified the first electrode(s) recruited in ictal activity for each individual seizure. In this study, we defined SOZ electrodes as any contact within 10 mm of these visually identified electrodes. We then calculated the rate of LoWS and IEDs during interictal and preictal periods for each electrode. Interictal epochs were sampled at least 2 hours away from any seizures. We then computed a 2-way analysis of variance (ANOVA) with region (SOZ vs outside SOZ) and periods of interest (preictal vs interictal) as factors. In a further analysis, we computed a 3-way ANOVA adding IEDs vs LoWS as the 3rd factor and Holm post hoc corrections. Note that when comparing the rate of LoWS between the preictal and interictal periods, we used the same threshold for detection of LoWS.

### Statistical Analyses

Statistical analyses were performed in Jamovi^33^ (version 2.5.4) and GraphPad Prism (version 10.0.0). Specific statistical tests are disclosed in individual sections. For *t* tests, we used the D'Agostino and Pearson test to establish the normality of data. Given the large sample size, this was not tested for ANOVA and LMM, but we verified and excluded outliers for these analyses, using the “Robust regression and Outlier removal” (ROUT) method implemented in GraphPad Prism, keeping default parameters (notably the coefficient Q = 1%). We used ANOVAs or LMMs for analyses that included ≥2 factors (mentioned in detailed models in respective method sections). Figures were designed using either Jamovi, GraphPad Prism, or Adobe Illustrator (v. 2023).

### Data Availability

Data are available on doi:10.18112/openneuro.ds004100.v1.1.3 under license CC0. Codes to detect IEDs and LoWS are in the following github link: github.com/bushlab-ucl/slowWaveDetection.

## Results

### Population Statistics

A total of 57 patients were included (30 women, mean age ±SD at recording 34 ± 10 years). We excluded 1 patient with a sampling frequency at 256 Hz (HUP173) to harmonize the analyses (given further), and 1 further patient had no interictal data available (HUP158), giving a total of 55 patients for subsequent analyses. For analyses of seizures, an additional 3 patients were excluded because they had unclear or no ictal activity. For analyses combining ictal and interictal activity, 52 patients were thus eventually included, with a total of 203 seizures. Intracranial EEG data from a total of 3,811 cortical contacts were analyzed (mean ± SD per patient: 69 ± 27, eFigure 1). Potential confounders were equally distributed between patients with successful and poor surgical outcome (Table 1). The Engel score for surgical outcome was available at 24 months for most patients (eTable 1). Further demographic details are presented in Table 1 and eTable 1.

Across patients and electrode channels, we identified a total of 141,398 LoWS associated with a downstate, occurring at a mean rate ±SD of 3.82 ± 0.95 minutes^−1^ (Figure 1, A and B), within the range found in our previous publication^20^ (3.6 ± 1.2 minutes^−1^). We also identified a total of 29,693 IEDs occurring at a rate of 0.71 ± 0.32 minutes^−1^ (Figure 1C). Visually, LoWS were different from IEDs (Figure 1, A and C and eFigures 2 and 3), and several previous observations argue that these 2 phenomena are distinct entities. Furthermore, using the amplitude, rising slope (from half-maximum to maximum amplitude), and descending slope (from maximum to half-maximum amplitude), we could cluster these 2 entities with an odds ratio to discriminate IEDs from LoWS that was significantly >1 (median [IQ]: 1.64 [1.26–3.17], one-sample Wilcoxon test, *p* < 0.0001, Figure 1D). This supports our assertion that IEDs and LoWS are distinct phenomena.

### Testing the Association Between the Incidence Rate of IEDs and Surgical Outcome

**Figure 2:**
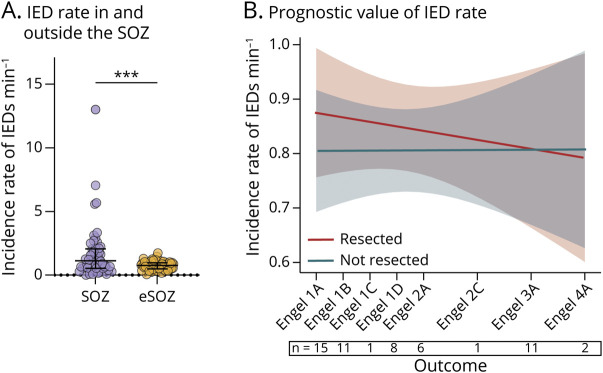
IEDs Localize the Seizure-Onset Zone, but Their Regional Incidence Is Not Associated With Surgical Outcome (A) The incidence rate of IEDs per electrode was higher inside the SOZ (1.13 [0.54–2.06] minute^−1^) than outside the SOZ (eSOZ; 0.76 [0.48–0.97] min-1; Wilcoxon test, *p* = 0.0006, n = 54 patients). This remained significant after removing outliers (SOZ: 0.86 [0.51–1.75] min-1, eSOZ: 0.76 [0.48–0.97] min-1; Wilcoxon test, *p* = 0.0054, n = 50 patients). (B) Linear mixed model (LMM) showing the incidence rate of IEDs min-1 in brain regions eventually resected (red) or not (blue) according to outcome. There was no interaction between outcome and resection status (F [1, 52.57] = 0.070, *p* = 0.793), indicating that the rate of IEDs is insufficient to identify brain regions that should be removed to optimize seizure freedom. Numbers below the x-axis indicate sample size per outcome. IED = interictal epileptiform discharge; SOZ = seizure-onset zone.

Extensive evidence has shown that IEDs are increased in the SOZ,^4,5^ but their ability to indicate which brain regions need to be removed to obtain seizure freedom is less clear.^6,26,34^ In line with this, we observed a significantly higher incidence rate of IEDs within vs outside the SOZ (within: 1.13 [0.54–2.06] minute^−1^ and outside: 0.76 [0.48–0.97] minute^−1^, Wilcoxon test: *p* = 0.0006, n = 54 patients, Figure 2A). We obtained similar results when selecting patients with outcome Engel 1, that is, those in whom the spatial delimitation of the SOZ is most reliable (within: 1.20 [0.57–1.91] minute^−1^ and outside: 0.74 [0.48–0.93] minute^−1^, Wilcoxon test: *p* = 0.0025, n = 34 patients). To test whether the IED rate was associated with surgical outcome, we used an LMM with IED rate as the dependent variable, resection status as a binary factor, and Engel score as an ordinal covariate. An association between IED rate and surgical outcome should be reflected in a statistical interaction between resection and Engel score, that is, a different rate in resected vs nonresected areas that varies across Engel stages. However, there was no outcome * resection status interaction (F [1, 52.57] = 0.070, *p* = 0.793, LMM, Omnibus test, Figure 2B). Hence, based on these data, the incidence rate of IEDs alone does not provide an accurate prognostic indicator.

### Testing the Association Between the Incidence Rate of LoWS and Surgical Outcome

**Figure 3:**
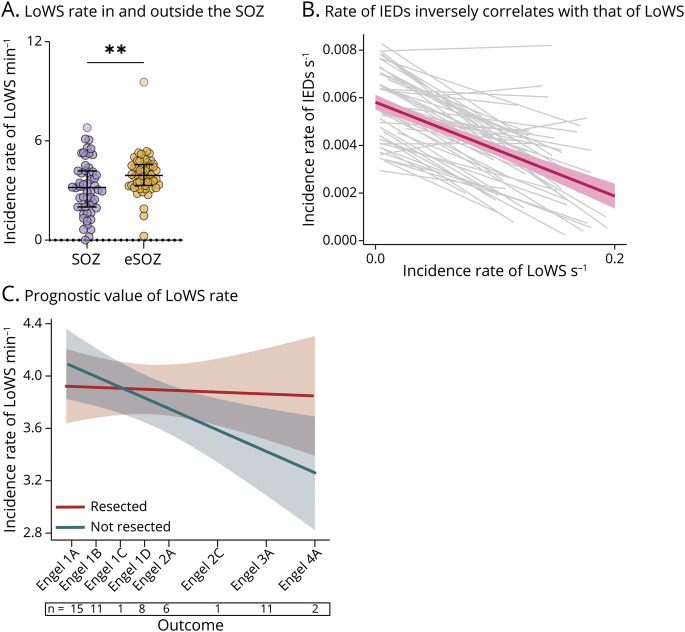
LoWS Are Rarer in the SOZ, but Their Regional Incidence Is Not Associated With Surgical Outcome (A) The incidence rate of LoWS per electrode was lower inside the SOZ (3.19 [2.01–4.19] min^−1^than outside the SOZ; eSOZ; 3.92 [3.31–4.56] minute^−1^; Wilcoxon test, *p* = 0.0017, n = 54 patients). This remained significant after removing outliers (SOZ: 3.19 [2.01–4.19] minute^−1^, eSOZ: 3.92 [3.31–4.54] minute^−1^; Wilcoxon test, *p* = 0.003, n = 52 patients). (B) Negative correlation between the rate second^−1^ of IEDs and that of LoWS. Each gray line represents 1 patient. The pink line represents the overall model, with standard error in light pink. (C) Linear mixed model (LMM) showing the incidence rate of LoWS minute^−1^ in brain regions eventually resected (red) or not (blue) according to outcome. There was no outcome * resection status interaction (F [1, 48.81] = 1.1032, *p* = 0.299), indicating that the rate of LoWS is insufficient to identify brain regions that should be removed to optimize seizure freedom. Numbers below the x-axis indicate sample size per outcome. IED = interictal epileptiform discharge; LoWS = Local Wake Slow Wave; SOZ = seizure-onset zone.

We then asked whether LoWS are a reliable marker of the SOZ and whether their incidence inside vs outside resected areas is associated with surgical outcome. In this case, we found a significantly lower incidence rate of LoWS within the SOZ (within SOZ, 3.19 [2.01–4.19] minute^−1^ than outside: 3.92 [3.31–4.56] minute^−1^, Wilcoxon test: *p* = 0.0017, n = 54 patients, Figure 3A). Again, this effect was conserved even when including only patients with outcome Engel I (within: 3.27 [2.47–4.15] minute^−1^ and outside: 4.15 [3.32–4.78] minute^−1^, Wilcoxon test: *p* = 0.0107, n = 34 patients). In addition, and in line with previous findings,^20^ we found a significant negative correlation between the rate of LoWS and that of IEDs across patients (F [1, 38.54] = 32.52, *p* < 0.001, LMM, Omnibus test, Figure 3B). However, this was not solely driven by the SOZ because including the SOZ as a binary factor in the LMM did not lead to a significant interaction with the rate of LoWS (F [1, 79.33] = 0.4179, *p* = 0.520, LMM, Omnibus test). We found no evidence that this effect was driven by the lobar location of the SOZ neither (eFigure 5).

Regarding the value of LoWS rate in gauging surgical outcome, and similar to findings related to the IED rate, there was no interaction between outcome and resection status (F [1, 48.81] = 1.1032, *p* = 0.299, LMM, Omnibus test, Figure 3C). This suggests that the incidence rate of LoWS—like IEDs—is insufficient to delineate the EZ.

### Incidence Rate of LoWS and IEDs Before Seizures

**Figure 4:**
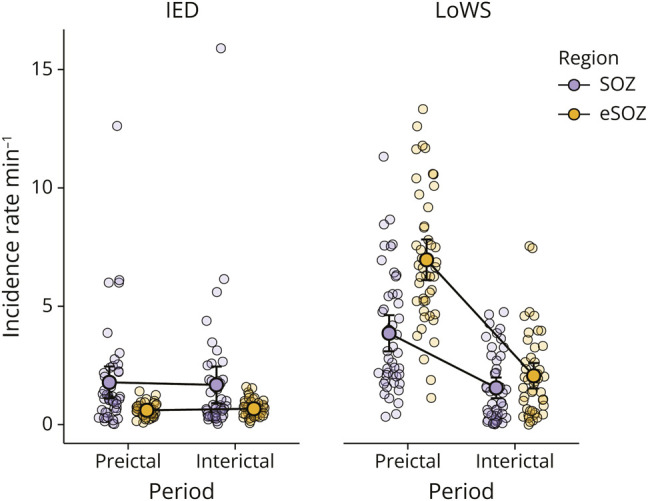
Rate of LoWS, but Not IED, Changes Before Seizures In 2 separate repeated-measures ANOVAs with 2 factors for IEDs and LoWS, respectively (region: SOZ vs extra-SOZ; period: interictal vs preictal), we observed only a main effect of region for IEDs (F [1, 44] = 11.237, *p* = 0.002) with no region * period interaction (F [1, 44] = 0.292, *p* = 0.592) and no main effect of period (F [1, 44] = 0.019, *p* = 0.892). Conversely, we found a significant region * period interaction for LoWS (F [1, 51] = 34.76, *p* < 0.001), as well as a main effect of region (F [1, 51] = 56.08, *p* < 0.001) and period (F [1, 51] = 54.46, *p* < 0.001). Post hoc analyses confirmed that the mean difference (preictal minus interictal) was higher outside (mean ± standard error: 4.53 ± 0.57 minutes^−1^) than inside (2.20 ± 0.41 minutes^−1^) the SOZ, and that both were significantly greater than zero (outside SOZ: *t* = 7.96, adjusted *p* < 0.001; inside SOZ: *t* = 5.34, adjusted *p* < 0.001). Then, using a repeated-measures ANOVA with 3 factors (region: SOZ vs extra-SOZ; event: IEDs vs LoWS; and period: interictal vs pre-ictal), we observed a significant region * event * period interaction (F [1, 44] = 27.441, *p* < 0.001), confirming the different trajectories of rates of IED and LoWS during interictal and preictal periods. n = 52 patients (n can differ depending on outliers). Bars indicate confidence intervals (hidden behind the means for the conditions IEDs, extra-SOZ in preictal and interictal periods). IED = interictal epileptiform discharge; LoWS = local wake slow wave; SOZ = seizure-onset zone.

The rate of IEDs has been shown to increase, decrease, or not change before seizures.^35-37^ To study the relationship between IED rate, rate of LoWS, and seizures, we ran separate repeated-measures 2-way ANOVAs for rates of IED and LoWS using period (interictal vs preictal) and region (SOZ vs extra-SOZ) as factors. We found a main effect of region for IEDs (F [1, 44] = 11.237, *p* = 0.002, Figure 4), but no main effect of period (F [1, 44] = 0.019, *p* = 0.892) or interaction (F [1, 44] = 0.292, *p* = 0.592). By contrast, LoWS showed a region * period interaction (F [1, 51] = 34.76, *p* < 0.001, Figure 4), as well as a main effect of region (F [1, 51] = 56.08, *p* < 0.001) and period (F [1, 51] = 54.46, *p* < 0.001). Hence, different patterns of change occur for LoWS and IEDs before seizures. To test whether these effects differed significantly between IEDs and LoWS, we next computed a 3-way ANOVA with LoWS vs IEDs as a further factor (hereafter named “event”). We found a significant region * event * period interaction (F [1, 44] = 27.441, *p* < 0.001, Figure 4), confirming that IEDs and LoWS follow different changes in incidence before seizures. Specifically, while the rate of LoWS increases before seizures, to a larger extent outside than inside the SOZ, the rate of IEDs is comparably greater inside vs outside the SOZ during both interictal and preictal periods (Figure 4).

### Temporal Interaction Between LoWS and IEDs

**Figure 5:**
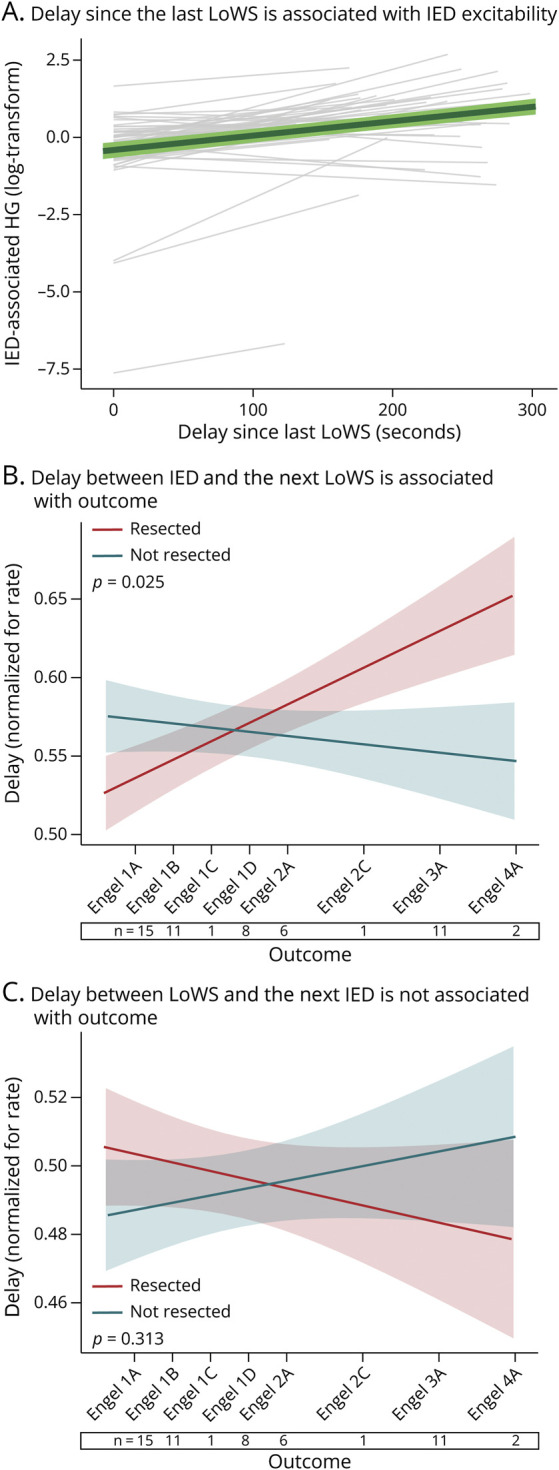
Relationship Between IEDs and LoWS Is Associated With Surgical Outcome (A) There is a significant association between IED-associated excitability and delay since the last LoWS (F [1, 39.56] = 30.15, *p* < 0.001, LMM, Omnibus test). Each gray line represents the relation between IED-associated excitability and delay since the last LoWS per patient. The green line represents the overall model with standard error in light green. (B) LMM showing the delay between IED and the next LoWS as a function of outcome and resection status. The significant resection status * Engel score interaction (F [1, 52.17] = 5.344, *p* = 0.025, LMM, Omnibus test) indicates that this delay is associated with surgical outcome. (C) LMM showing the delay between LoWS and the next IED as a function of outcome and resection status. The lack of resection status * Engel score interaction (F [1, 52.56] = 1.038, *p* = 0.313, LMM, Omnibus test) indicates that this delay is not indicative of surgical outcome. For (B) and (C), numbers below the x-axis indicate sample size per outcome. IED = interictal epileptiform discharge; LMM = linear mixed model; LoWS = local wake slow wave.

Previously, we described a relationship between time since the last LoWS and network excitability during the subsequent IED, as estimated by HG activity.^20^ In this study, using a separate and larger cohort, we replicated this finding; the longer the delay since the last LoWS, the higher the IED-associated HG (F [1, 39.56] = 30.15, *p* < 0.001, LMM, Omnibus test, Figure 5A). Next, we tested whether the delay between IEDs and LoWS (or vice versa) differs inside vs outside the EZ, that is, whether this dynamic relationship has any prognostic value. To do so, we corrected the time delay between events to account for differences in rates of LoWS and IED across patients and electrodes (Methods) and then used this “normalized” delay as the dependent variable in an LMM. If delay had any prognostic value, then its difference between resected and nonresected areas should vary across Engel scores. We, therefore, tested whether this LMM revealed a significant statistical interaction between resection status and Engel score. We found that the delay from each IED to the next LoWS is associated with surgical outcome (F [1, 52.17] = 5.344, *p* = 0.025, LMM, Omnibus test, Figure 5B). Specifically, short delays were found in resected areas of patients with successful outcome and in nonresected areas of patients with poor outcome. As a way to control for the topographical variability of the SOZ, lesional status, and type of resection, we computed another LMM with SOZ; presence, or not, of a lesion; and type of resection (eTable 1) as further random coefficients and found that IED-to-LoWS delays was associated with surgical outcome (F [1, 103] = 5.97, *p* = 0.02). Similar results were obtained using the International League Against Epilepsy (ILAE) score (F [1, 52.20] = 6.314, *p* = 0.015, LMM, Omnibus test). By contrast, long delays were found in nonresected areas of patients with successful outcome and in resected areas of patients with poor outcome. Conversely, the delay from each LoWS to the next IED was not associated with outcome (F [1, 52.56] = 1.038, *p* = 0.313, LMM, Omnibus test, Figure 5C).

### Association Between IED-to-LoWS Delay and Surgical Outcome

**Figure 6:**
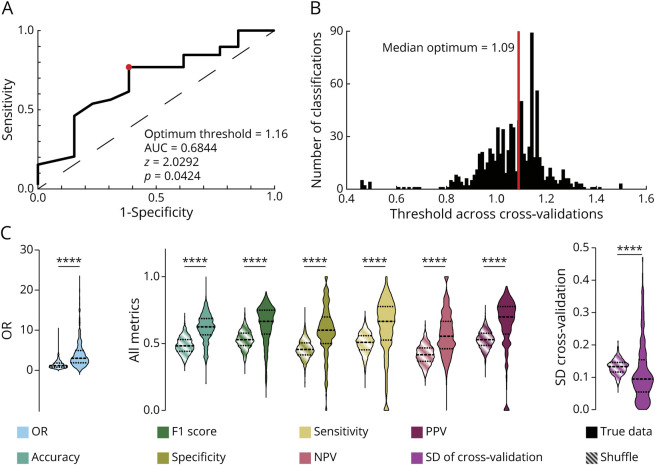
Classification Analysis Confirms That IED-to-SW Delay Is Associated With Outcome (A) Receiver Operating Characteristic (ROC) using the entire data set shows a significant area under the curve (AUC = 0.68, *p* = 0.04). (B) Using a classification algorithm (Methods), we identified an optimal threshold close to that anticipated by the ROC curve in (A). (C) The classification on the validation set confirmed that all parameters were above chance level (odds ratio: 3 [1.88–4.94]; accuracy: 0.63 [0.57–0.69]; F1 score: 0.67 [0.57–0.75]; specificity: 0.6 [0.5–0.7]; sensitivity: 0.67 [0.53–0.78]; negative predictive value: 0.56 [0.46–0.67]; positive predictive value: 0.7 [0.57–0.78]). In particular, the model reached an accuracy of 63%. Note that the model also showed less variability than expected by chance, as indicated by the low SD of the threshold for discriminating good from poor outcomes (violet violin plot, 0.09 [0.05–0.15], *p* < 0.0001, bootstrap; also refer to eFigure 4), supporting the robustness of the classifier. IED = epileptiform discharge; NPV = negative predictive value; OR = odds ratio; PPV = positive predictive value.

To test whether the population differences observed above hold on a patient-by-patient basis, we next attempted to classify patient outcomes based on the IED-to-LoWS delay (Methods, Figure 6, and eFigure 4). Specifically, we calculated the corrected delays of IEDs to LoWS (and vice versa) in resected and nonresected areas, and the ratio (resected divided by nonresected) was used as a metric to identify patients with good vs poor outcome. First, using all patients, we obtained a significant area under the receiver operating curve of 0.68 (z = 2.03, *p* = 0.04, Figure 6A). We found an optimal threshold below (respectively above) which regions are likely to be resected (respectively nonresected) to optimize outcome (Figure 6B). All metrics of performance were significantly above chance level (Figure 6C). In particular, we obtained a median accuracy of 0.63. This was significantly higher than the accuracy obtained using IEDs alone, that is, one of the most classical epileptic biomarker,^4^ for which we obtained a median accuracy of 0.52 (Mann-Whitney test, *p* < 0.0001). Last, we also observed a significant classification using Engel score I as good outcome, for which the classification accuracy reached 0.6 (significantly above chance level, Mann-Whitney test, *p* < 0.0001). Altogether, this indicates that the IED-to-LoWS delay is associated with outcome.

## Discussion

In this study, we observed that IED-to-LoWS delay is associated with surgical outcome in patients with drug-resistant epilepsy, despite the fact that each variable (i.e., LoWS and IED) taken individually is not. Performance is similar to that of more complex models,^38^ which makes these results particularly encouraging, but we do not argue that our metric can replace a thorough preoperative assessment of epilepsy. Rather, we propose that IED-to-LoWS delay constitutes a further helpful biomarker in presurgical evaluations, similar to other candidate biomarkers of the EZ, such as high-frequency oscillations.^39^ This is particularly timely, because the development of new biomarkers to refine the delimitation of the SOZ is an active field of research.^4-7,13,14,22,29,34^

Delays were measured relative to the median across resected and nonresected areas. In the workup of presurgical evaluation of epilepsy, one could follow the same approach and test how these delays vary across different combinations of recording contacts that are targeted or not for resection. An alternative would be to first identify, using a classification approach, a threshold below which a specific electrode should be considered for resection. Larger data sets composed of longer recordings and thus more IEDs will be necessary to capture reliable regression coefficients on an electrode level.

In line with the restorative function of sleep SWs,^40-43^ we had shown in previous work that the LoWS-to-IED delay correlates with excitability during that IED.^20^ In the current study, the LoWS to next IED delay was not associated with outcome while short IED-to-LoWS delays were found in the EZ. This is consistent with the hypothesis that LoWS are triggered in response to increased excitability and with the observation of an increased incidence rate of LoWS before seizures (Figure 4). It also implies that while LoWS regulate the excitability within the SOZ, the generation of LoWS is governed by increases in network excitability.

Is there a candidate mechanism that could explain our observations? During sleep, evidence suggests that SWs participate in the normalization of neuronal excitability^44-47^ and a downregulation of alpha-amino-3-hydroxy-5-methyl-4-isoxazolepropionic acid (AMPA) receptors on burst firing has been identified as a possible mechanism.^40,45^ The shorter latency between IED and LoWS in the EZ could reflect decreased inhibition of that region, leading to LoWS occurring closer to preceding IEDs. Although the classifier dissociated patients with good from poor outcomes, its sensitivity and specificity—as for any other classifier—were not 100%. This could be due to inherent noise but may also suggest that, in specific regions, a short delay reflects a beneficial, compensatory mechanism. A control study, which would need a much larger data set, given the limited spatial sampling of each patient, would be to identify these discrepant regions and see whether they are consistent across patients.

Increases, decreases, and no change in IED rates have been observed before seizures.^35-37,48^ These differing observations could be due to different subtypes of IEDs, which have different associations with seizure risk.^49,50^ In our data, we did not identify an association between IED rates in the interictal vs preictal period. Of interest, our findings related to LoWS are more complex. In the preictal period, LoWS rates increased, more so outside the SOZ, with remarkably large effect sizes (Figure 4). The lower increase in the rate of LoWS inside the SOZ could also reflect a dynamic disruption of the beneficial effect of LoWS. Time-resolved analyses of rates of IEDs and LoWS will provide further understanding of the relationship between these entities and their potential impact on seizures.

Although we used a data set of 57 patients for this study, larger studies with multicenter data will be required to validate these results and to determine in more depth the impact of etiology. This is particularly true given the limited spatial sampling of intracranial EEG. However, although this is a limitation, our observations are expected to generalize across other populations if we assume that the same spatial suboptimal sampling, with balanced proportion of contacts within and outside the EZ, occurs in all patients evaluated for epilepsy surgery. Future studies on multimodal analysis, combining imaging and electrophysiology, are also necessary to clarify the potential role of underlying pathology (e.g., sclerosis and dysplasia). We had shown in a previous work that LoWS replicate core features of sleep SWs, including the downstate of neuronal activity.^20^ However, further work is necessary to clarify the extent of both mechanistic and morphological similarities between these wake and sleep entities in detail. This would help to refine any biomarker associated with epileptic activity. Last, although our metric may seem straightforward, it relies on the automatic detection of IEDs and LoWS, which involves computation of decreases in HG power. Hence, this complexity might pose a challenge that will need to be simplified for practical applicability.

In sum, our work confirms a recently identified beneficial effect of LoWS on IED-associated excitability. It further demonstrates that the preictal period is marked by spatially specific changes in the incidence rate of LoWS and reveals a new candidate biomarker associated with outcomes of epilepsy surgery.

## Glossary

EZ: epileptogenic zone
HG: high-γ
HUP: Hospital of the University of Pennsylvania
IED: interictal epileptiform discharge
LMM: linear mixed model
LoWS: local wake slow waves
NINDS: National Institute of Neurological Disorders and Stroke
SOZ: seizure-onset zone
